# Phytochemical Profiles and Antioxidant Activities in Six Species of Ramie Leaves

**DOI:** 10.1371/journal.pone.0108140

**Published:** 2014-09-22

**Authors:** Yongsheng Chen, Gaoyan Wang, Hong Wang, Chaohua Cheng, Gonggu Zang, Xinbo Guo, Rui Hai Liu

**Affiliations:** 1 School of Light Industry and Food Sciences, South China University of Technology, Guangzhou, Guangdong, The People's Republic of China; 2 Department of Food Science, Cornell University, Ithaca, New York, United States of America; 3 Institute of Bast Fiber Crops, Chinese Academy of Agricultural Sciences, Changsha, Hunan, The People's Republic of China; 4 Department of Food Science and Institute of Comparative and Environmental Toxicology, Stocking Hall, Cornell University, Ithaca, New York, United States of America; University of Sassari, Italy

## Abstract

Increased consumption of vegetables or plant food has been associated with decreased risk of developing major chronic diseases, such as cancers, diabetes, cardiovascular diseases, and age-related functional decline. Ramie leaves are rich in phenolics and flavonoids, which have been suggested for human health benefits. Phenolic contents, flavonoid contents, phenolic compounds, and anti-cancer properties in six species of ramie leaves were analyzed by Folin-reagent method, sodium borohydride/chloranil-based assay (SBC), HPLC method and antiproliferation, cytoxicity, respectively. Antioxidant activities were measured through peroxyl radical scavenging capacity (PSC) method, oxygen radical absorbance capacity (ORAC) method, and cellular antioxidant activity (CAA). Research indicated that *Boehmeria penduliflora* contained the highest total phenolic content (2313.7±27.28 mg GAE/100 g FW), and flavonoid content (1682.4±27.70 mg CAE/100 g FW). *Boehmeria tricuspis* showed the highest PSC value (9574.8±117.63 µM vit. C equiv./100 g FW), while *Boehmeria penduliflora* indicated the highest ORAC value (330.44±16.88 µmol Trolox equiv./g FW). The antioxidant activities were correlated with phenolic contents and flavonoid contents. *Boehmeria tricuspis* had the highest antiproliferative capacity with the lowest EC_50_ (4.11±0.19 mg/mL). The results for the analyzed ramie for CAA were significantly different from each other (*p*<0.05), *Boehmeria tricuspis* had the highest CAA value (133.63±7.10 µmol QE/100 g). Benzoic acid, 4-coumaric acid, caffeic acid, and ferulic acid were the dominant phenolic ingredients in the ramie leaves according to HPLC analysis. Our research is the first report to study the phytochemical profiles and antioxidant activities in different species of ramie leaves for their health benefit.

## Introduction

Phytochemicals are a class of bioactive non-nutrient compounds which usually exist in fruits, vegetables, grains, and other based-plant foods. Phytochemicals have been linked to reducing the risk of major chronic diseases [1]. Phytochemicals have potential antioxidant activity and are able to scavenge hydroxyl radicals, capture peroxyl radicals, inhibit hydrogen peroxide, and quench reactive nitrogen species [2]–[4]. Some phytochemicals have been proved to show higher antioxidant activities than vitamin C and vitamin E [3]. It is common that different plants contain different types of phytochemicals with disparate structures and protective mechanisms, and demonstrate different level of antioxidant activities.

Free radicals and other reactive oxygen species are constantly generated in human cells and organisms as a result of aerobic metabolism, some of which are necessary for life. Their pivotal role is to maintain balance between oxidants and antioxidants to maintain optimal physiological conditions [1]. However, excessive of oxidants may cause oxidative stress and lead to unbalanced physiological conditions. Oxidative stress could cause oxidative damages to large bio-molecules (lipids, proteins, and DNA), eventually resulting in development of many chronic diseases, such as cancer, cardiovascular, and neurodegenerative diseases [5]. Antioxidants are effective substances, which can protect the tissue from oxidative damage by modulating the effects of reactive oxidants [4]. Therefore, natural antioxidants from plant extracts have attracted increasing interests due to consumer concern about the safety of the synthetic antioxidants. Phenolics and flavonoids are biologically active plant compounds, widely spread in fruits, vegetables, teas, and corns, and have also attracted extensive research interests due to their strong antioxidant activities. Phenolics and flavonoids are the secondary metabolites of plants, which is very important in maintaining essential functions in the reproduction and growth of the plants [6], and flavonoids are a kind of phenolics.

Ramie, widely cultivated in China and a member of the *Urticaceae* family *Bochmeria*, is a perennial herbaceous plant commonly grown for its fibers. Ramie has vigorous vegetative growth and can be harvested three times per year in south China areas. Ramie leaves is a abundant by-product and have been consumed in the form of rice-cake or as a tea and are used in traditional medicine for treatment of diarrhea and snake bites [7]. Ramie extracts have high-grade of physiological functions, such as antioxidant, antibacterial and antifungal activities that restrain both bacterial and fungal attacks, and also anticancer effects on lung and liver cancers [8]. Ramie leaves is not only good sources for dietary and medical use; it is also excellent sources for phytochemicals, such as phenolics and flavonoids. They have been reported to show higher antioxidant activity than ordinary diet[9]. Therefore, exploitation for potential value-add usage of those parts would be necessary. In present research, we analyzed and compared the phytochemical profiles (phenolic content, flavonoid content and phenolic compounds) and antioxidant activities of six species of Ramie for evaluating its potential application as health beneficial nutraceuticals.

## Materials and Methods

### Chemicals

Ascorbic acid (ASA), 2,6-dichloroindophenol sodium salt hydrate, chloranil, catechin hydrate, vanillin, Folin-Ciocalteu reagent, hydrocortisone, penicillin, streptomycin, gentamicin, Gallic acid, 2,2′-Azobis-amidinopropane (ABAP) and dichlorofluorescin diacetate (DCFH-DA) were purchased from Sigma Chemical Co. (St. Louis, MO, USA). NaOH, KH_2_PO_4_, KOH, AlCl_3_, acetic acid, NaHCO_3_, and K_2_HPO_4_ were bought from Sangon (Shanghai, China). Chlorogenic acid, Caffeic acid, 4-Coumaric acid, Ferulic acid and benzoic aicd, NaBH_4_ were bought from Aladdin Co. (Shanghai, China). WME medium, Hank's balanced salt solution (HBSS), epidermal growth factor, heparin, insulin, and other cell culture reagents were purchased from Gibco Biotechnology Co. All reagents used were of analytical grade.

### Plant materials

Six species of fresh ramie leaves (Boehmeria nivea, Boehmeria longispica, Boehmeria clidemioides, Boehmeria macrophylla, Boehmeria tricuspis, Boehmeria penduliflora) were procured from Institute of Bast Fiber Crops (Changsha, China). All samples were individually mixed thoroughly and were stored at −40°C until use.

### Extraction of soluble free phytochemicals

Soluble free phytochemicals of ramie leaves were prepared using the modified method as previously reported [10], [11]. Briefly, 20 g of samples were first blended in a blender using 150 mL of chilled 80% acetone (1∶7.5, w/v) for 3 min. Samples were subsequently homogenized with a IKA homogenizer for another 3 min. Second, the homogenates were centrifuged at 3675 g for 10 min, 4°C. Supernatants were filtered with Whatman no. 2 filter paper, and the filtrate was collected and evaporated at 45°C until 10% of the filtrates had been retained. The filtrates were recovered with water to a final volume of 10 mL and stored at −80°C until further analysis. All the extractions were performed in triplicates.

### Extraction of bound phytochemicals

Bound phytochemicals in ramie leaves were extracted using the modified methods as previously reported [10], [11]. Briefly, the filter residues from above soluble free extraction were collected, flushed with nitrogen gas, sealed, and hydrolyzed directly with 20 mL of 4 M NaOH at room temperature for 1 hour with shaking. The mixture was acidified with concentrated hydrochloric acid to pH 2, and extracted ten times with ethyl acetate. The ethyl acetate fractions were evaporated at 45°C under vacuum to dryness. Bound phytochemicals were reconstituted in 10 mL water, aliquoted to 1 mL, and stored at −40°C until analysis.

### Determination of total phenolic content

Total phenolic content in ramie leaves were determined using the Folin-Ciocalteu colorimetric method with modification [12], [13]. In brief, standard curve using gallic acid (0.0–600.0 µg/mL) was made. For each analysis, 100 µL of the standard gallic acid solution or extracts sample were added to 0.4 mL of water in each test tube. Folin-Ciocalteu reagent (0.1 mL) was added to the solution and allowed to react for 6 min to ensure that the Folin-Ciocalteu reagent reacted completely with the oxidizable phenolates in the sample. Second, 1 mL of 7% sodium carbonate solution was added to neutralize the reaction, and 0.8 mL of deionized water was added into the test tubes to adjust the final volume to 2.4 mL. The samples were mixed and allowed to stand for 90 min at room temperature. The absorbance was measured at 760 nm after the color was developed by DU 730 Nucleic Acid/Protein Analyzer (BECKMAN, USA). The absorbance values were calculated based on the standard curve of known gallic acid concentrations and expressed as milligram of gallic acid equivalents (GAE) per 100 g fresh weight (FW). Data were reported as mean ± SD for triplicates.

### Determination of water-soluble flavonoid content

The total flavonoid content were determined using the sodium borohydride/chloranil (SBC) method [14]. Briefly, 1 mL of phytochemical extracts of tested samples was added into test tubes (15×150 mm), placed under nitrogen gas to dryness, and reconstituted in 1 mL of terahydrofuran/ethanol (THF/EtOH, 1∶1, v/v). Catechin hydrate standard (0.3–10.0 mM) was prepared fresh in 1 mL of THF/EtOH (1∶1, v/v). Each test tube with sample or standard was added with 0.5 mL of 50 mM NaBH_4_ solution and 0.5 mL of 74.6 mM AlCl_3_ solution. Subsequently, the test tubes were shaken in an orbital shaker at 180 rpm at room temperature for 30 min. An additional 0.5 mL of 50.0 mM NaBH_4_ solution was added into each test tube with shaking continued for another 30 min at room temperature. Chilled 2.0 mL of 0.8 M acetic acid solution was added into each test tube and kept in the dark for 15 min after being thoroughly mixed. Then, 1 mL 20.0 mM chloranil was added in each tube and heated at 99°C with shaking for 60 min. The reaction solutions were cooled using tap water, and the volume was brought to 4 mL using methanol. In the next step, 1 mL of 16% (w/v) vanillin was added into each tube and mixed. 2 mL of 12 M HCl was subsequently added into each tube and kept in the dark for 15 min after a thorough mix. The reaction solutions were centrifuged at 2500 g for 10 min. The absorbance of the mixture was immediately measured at 490 nm against a prepared blank using a DU 730 Nucleic Acid/Protein Analyzer (BECMAN, USA). Results were calculated by using the standard curve of catechin hydrate concentration. Total flavonoid content was expressed as milligram of catechin equivalents (CAE) per 100 g of fresh weight of ramie. Results were reported as mean ± SD in triplicates.

### Hydrophilic peroxyl radical scavenging capacity (PSC) assay

Total antioxidant activity was measured using the hydrophilic peroxyl radical scavenging capacity (Hydro-PSC) assay [2]. Ascorbic acid and phytochemical extracts were diluted in appropriate concentration by using 75 mM phosphate buffer (pH 7.4). Ascorbic acid was prepared fresh and diluted to 6.3, 4.8, 3.2, 2.4, and 1.0 µg/mL. Gallic acid was made fresh and diluted to 5.5, 3.5, 2.7, 1.4, and 0.9 µg/mL. The reaction mix contained 75 mM phosphate buffer at pH 7.4, 40 mM ABAP, 13.26 µM DCFH dye, and the appropriate concentrations of the pure antioxidant compound or sample extracts. The dye was prehydrolyzed with 1 mM KOH to remove the diacetate moiety just prior to use in the reaction, and the reaction was carried out at 37°C, in a total volume of 250 µL using a 96-well plate. Fluorescence generation was monitored (excitation at 485 nm and emission at 535 nm) with a Fluoroskan Ascent fluorescent spectrophotometer (Molecular Devices, USA). Data were acquired with the Ascent Software, version 2.6 (Molecular Devices, USA) running on a PC. The areas under the fluorescence reaction time kinetic curve (AUC) for both control and samples were integrated and used as the basis for calculating peroxyl radical scavenging capacity (PSC) using the equation PSC (value)  = 1 − (SA/CA), where SA is AUC for the sample or standard dilution and CA is AUC for the control reaction. Compounds or extracts inhibiting the oxidation of DCFH produced smaller SA and higher PSC values. The parameter EC_50_ was defined as the dose required to cause a 50% inhibition (PSC unit  = 0.5) for each pure compound or sample extract, and was used as the basis for comparing the antioxidant activities of different compounds or samples. Results obtained for antioxidant activities of sample extracts were presented as micromole of vitamin C equivalents per 100 g of sample ± SD with triplicates.

### Measurement of oxygen radical scavenging capacity (ORAC)

The peroxyl radical scavenging efficacy of selected ramie extracts was determined using the ORAC assay described by Huang et al. [15] and modified in our group [16]. Briefly, 20 µL of blank, Trolox standard, or ramie leave extract dilutions with 75 mM phosphate buffer, pH 7.4 (working buffer), were added to triplicate wells in a blank, clear-bottom, 96-well microplate (Corning Scientific), and incubate at 37°C for 10 min. The outside wells of the plate were not used as there were much more variation from them than the inner wells. In addition, the triplicate samples were distributed throughout the microplate and were not placed side-by-side, to avoid any effects on readings due to location. 200 µL of 0.96 µM fluorescein in working buffer was added to each well and incubated at 37°C for 20 min. Then add 20 µL of freshly prepared 119.4 mM ABAP in working buffer to each well. The microplate was immediately measured using a Fluoroskan Ascent FL plate-reader (Molecular Devices, USA) at 37°C. The decay of fluorescenece at 535 nm was measured with excitation at 485 nm for 35 cycle every 4.5 min. The areas under the fluorescence versus time curve for the samples minus the area under the curve for the blank were calculate and compared to a standard curve of the areas under the curve for 6.25, 12.5, 25, and 50 µM Trolox standards minus the area under the curve for blank. ORAC values were expressed as µmol Trolox equiv./g FW ± SD for triplicate data from one experiment.

### Analyses of phenolic acid compounds by HPLC

The chromatographic analyses were performed according to Malta et al. [17], with slight modifications in our laboratory. Briefly, phenolic acids were determined on a Waters (Waters Corp, Milford, MA) HPLC system, consisting of a Waters 1525 HPLC pump (Waters Corp, Milford, MA), one Waters 2998 dual-wavelength absorbance detector and an intelligent sampler 2707. The chromatographic data were recorded and processed by Waters software. A Waters C_18_ column (5 µm, 250 mm×4.6 mm) was employed for the separation of ramie extracts. The flow rate of the mobile phase was 1 mL/min. Mobile phase A was water with 0.02% TFA, and phase B was methanol with 0.02% TFA. The gradient conditions were as follows: 0–5 min, 25% B; 5–10 min, 25–30% B; 10–16 min, 30–45% B; 16–18 min, 45% B; 18–40 min, 45–80% B; 40–50 min, 80–25% B. The detection wavelength was 270 nm. Authentic standards of acids included ferulic acid, chlorogenic acid, caffeic acid, 4-coumaric acid and benzoic acids in methanol. The recovery of caffeic acid, chlorogenic acid, 4-coumaric acid, ferulic acid and benzoic acid were 100.1±0.11%, 98.7±0.72%, 101.5±0.02%, 101.2±0.03% and 99.9±0.26% from the phytochemical extracts, respectively. The results were expressed as milligram per 100 g of fresh weight according to the standard curve. Results obtained for sample extracts were expressed as mean ± SD for triplicates.

### Cell culture

Human liver cancer cell line HepG2, purchased from ATCC company (ATCC HB-8065), was grown in growth medium (WME supplemented with 5% FBS, 10 mM Hepes, 2 mM L-glutamine, 5 µg/mL insulin, 0.05 µg/mL hydrocortisone, 50 units/mL penicillin, 50 µg/mL streptomycin, and 100 µg/mL gentmycin), were maintained at 37°C and 5% CO_2_.

### CAA of ramie leaves extracts

The CAA assay has been previously described [18] and modified by our lab. Briefly, HepG2 cells were seeded at a density of 6×10^4^ cells/well on a 96-well microplate in 100 µL of growth medium/well. About twenty-four hours postseeding, the growth medium was removed and the wells were washed with PBS. Microplate were treated for 1 h with 100 µL of medium containing ramie extracts plus 50 µM DCFH-DA. Certain wells were washed with 100 µL of PBS (i.e., PBS wash protocol) and certain wells were not washed (i.e., no PBS wash protocol). PBS wash protocol means that cells are pretreated with ramie extract before the ABAP is added; on the other hand, no PBS wash means that cells are cotreated with ramie extract and ABAP. ABAP (600 µM) in 100 µL of HBSS was added to the cells. The 96-well microplate was placed in a Fluoroskan Ascent fluorescent spectrophotometer (SoftMax systems, Molecular Devices, US) at 37°C. The emission wavelength at 535 nm was measured after an excitation at 485 nm every 5 min for 1 h.

### Quantification of CAA

After the subtraction of the blank and the initial fluorescence values, the area under the fluorescence versus time curve was calculated to determine the CAA value at each ramie extract concentration. The following equation was used: CAA (units)  = 1–(□ SA/□ CA) where □ SA is the integrated area under the sample in the fluorescence versus time curve, and □ CA is the integrated area under the control in the flurescence versus time curve. The median effective dose (EC_50_) of the ramie extracts was calculated from the median effect plot of log (f_a_/f_u_) versus log (dose), where f_a_ is the fraction affected by the treatment (CAA unit) and fu is the fraction unaffected (1- CAA unit) by the treatment. The EC_50_ values were expressed as the mean ± SD using triplicate data sets obtained from the same experiment. EC_50_ values were converted to CAA values, which were expressed as micromoles of quercetin equivalents (QE) per 100 g of ramie, using the mean EC_50_ value for quercetin from three separate experiments.

### Cell proliferation inhibiting test

The antiproliferative effects of ramie extracts were assessed in HepG2 cell methylene blue colorimetric method [19]. Briefly, 100 µL of PBS was added to the peripheral wells of the 96-well microplate, and 100 µL of HepG2 cell suspension was seeded at a density of 2.5×10^4^/well in the central wells of the 96-well microplate. Incubate the cells for 4 h at 37°C in 5% CO_2_ to allow cells to sufficiently attach. Remove the growth medium from the central wells, and 100 µL of fresh medium containing different concentrations of ramie extracts was added. The wells receiving cell suspension without ramie extract served as the control. The plates were incubated for 72 h at 37°C. Following the incubation, the staining was removed, and the 96-well microplates were washed six times in deionized water until the water was clear. Then 100 µL of elution buffer (49% PBS, 50% ethanol, and 1% acetic acid) was added to each well. The 96-well microplates were transferred to a table oscillator for 20 min. Absorbance was measured at 570 nm using a microplate reader. Each sample was measured at least three times. The anti-proliferative effects were assessed by the IC_50_ values, which were expressed as milligrams of ramie extracts per milliliter.

### Cytotoxicity test

A cytotoxicity test was performed using the modified methylene blue assay [19], [20]. Briefly, HepG2 cells were seeded at a density of 4×10^4^ cells/well on a 96-well microplate with 100 µL of growth medium/well. The cells were incubated for 24 h at 37°C and 5% CO_2_. After the cells had attached to the wells, the growth medium was removed and the cells were washed with PBS. Then 100 µL of medium with different concentrations of ramie extract was added to each well; wells that received medium without ramie extract served as the control. After 24 h of incubation at 37°C, the medium was removed and the wells were washed with PBS. Add 50 µL of methylene blue solution (98% HBSS, 0.67% glutaraldehyde, and 0.6% methylene blue) to each well of the culture plate. Then incubate plate at 37°C for 60 min. Remove the methylene blue solution and rinse plate in water until the water was clear. Subsequently, add 100 µL of elution buffer (49% PBS, 50% ethanol, and 1% acetic acid) to each well with multiple channel pipette. Place well on a plate rotator at room temperature for 20 min, and read plates using a microplate reader at 570 nm wavelength. Concentrations of ramie extract that decrease the absorbance by > 10% when compared to the control are considered to be cytotoxic.

### Statistical analysis

Statistical analyses were performed using Sigmaplot software 11.0 (Sustat Software, Inc., Chicago, IL) and dose-effect analysis was performed using Calcusyn software version 2.0 (Biosoft, Cambridge, U.K.). Results were subjected to ANOVA calculated using SPSS software 21 (SPSS Inc., Chicago, IL, USA) and differences between means were located using Tukey's multiple comparison test. Significance was determined at *p*<0.05. All data were reported as mean ± SD for triplicates.

## Results

### Phenolic content in ramie leaves

**Figure 1:**
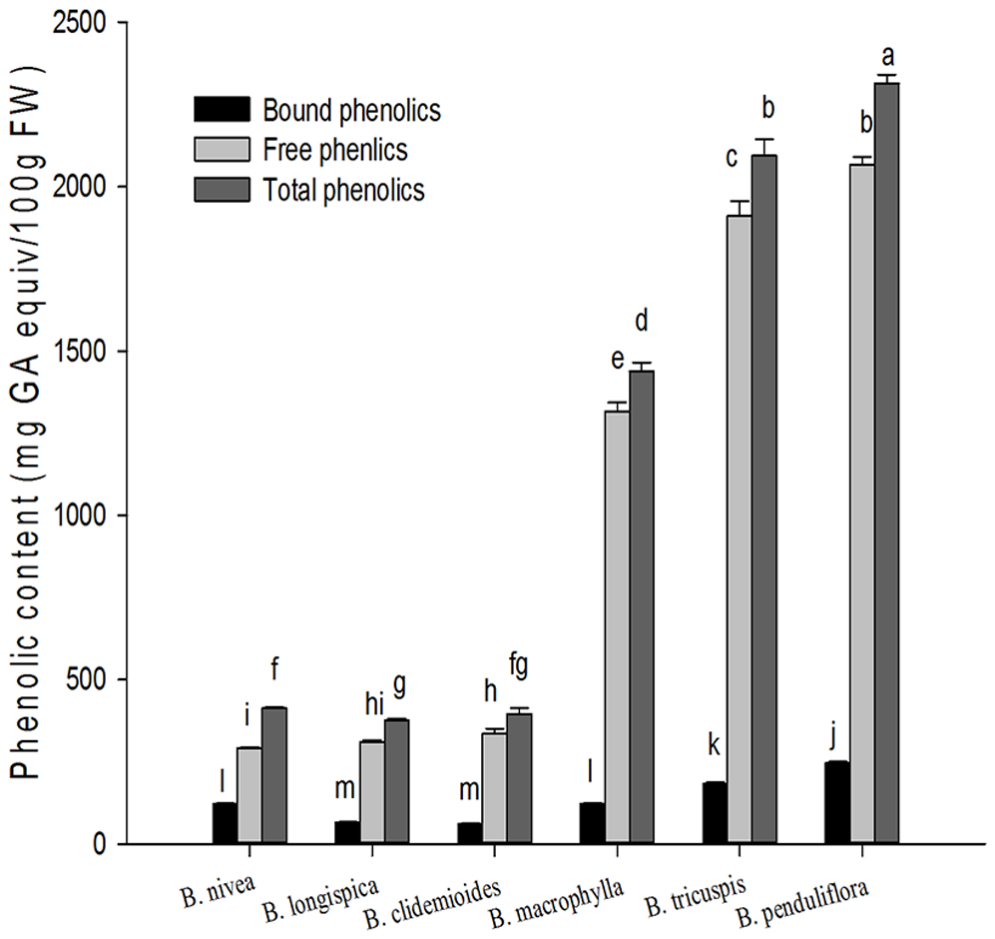
Phenolic contents in six species of ramie leaves (mean ± SD, n = 3). Bars with no letters in common are significantly different (*p*<0.05).

The total phenolic content of ramie leaves are presented in Figure 1. The free phenolic contents of six tested ramie samples ranged from 291.44 (*B. nivea*) to 2067.4 (*B. ppenduliflora*) mg of GAE/100 g of FW. The bound phenolic contents of six tested ramie samples ranged from 60.43 (*B. clidemioides*) to 246.38 (*B. penduliflora*) mg of GAE/100 g of FW. The percentage of bound phenolics to the total ranged from 8.52 (*B. macrophylla*) to 29.48% (*B. nivea*). The total phenolic contents of six tested ramie samples ranged from 376.08 (*B. longispica*) to 2313.7 (*B. penduliflora*) mg of GAE/100 g of FW. *B. penduliflora* leaves had the highest total phenolics (2313.7±27.28 mg of GAE/100 g of FW). *B. penduliflora* leaves had the highest free phenolics (2067.4±22.74 mg of GAE/100 g of FW), up to 89.35% of total phenolics, and had the highest bound phenolics (246.38±4.59 mg of GAE/100 g of FW), accounted for 10.65% of total phenolics.

### Flavonoid content in ramie leaves

**Figure 2:**
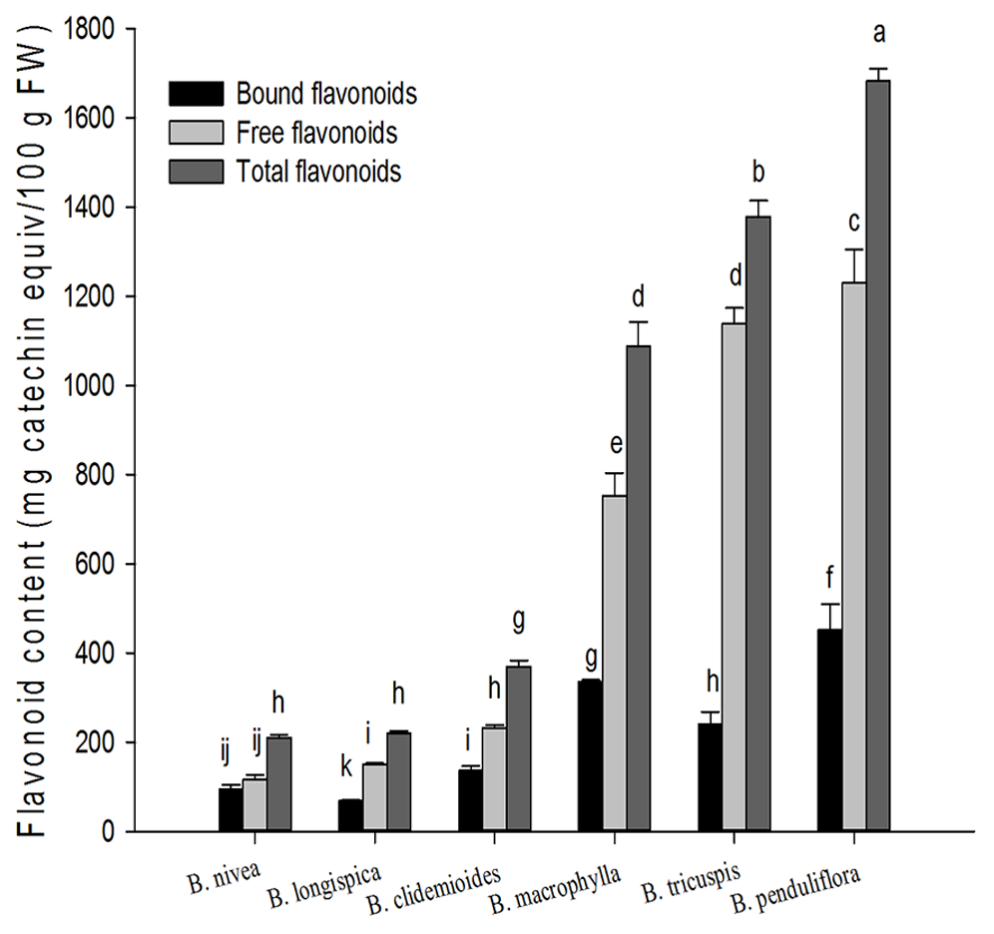
Flavonoid contents in six species of ramie leaves (mean ± SD, n = 3). Bars with no letters in common are significantly different (*p*<0.05).

Total flavonoid content of ramie leaves are presented in Figure 2. The free flavonoid contents of six tested ramie samples ranged from 116.34 (*B. nivea*) to 1229.8 (*B. penduliflora*) mg of CAE/100 g of FW. The percentage contribution of free flavonoid to the total ranged from 55.29 (*B. nivea*) to 82.57% (*B. tricuspis*). The bound flavonoid contents of six tested ramie samples ranged from 69.09 (*B. longispica*) to 452.63 (*B. penduliflora*) mg of CAE/100 g of FW. The percentage contribution of bound flavonoid to the total ranged from 17.43 (*B. tricuspis*) to 44.71% (*B. nivea*). The total flavonoid contents of six tested ramie samples ranged from 210.41 (*B. nivea*) to 1682.4 (*B. penduliflora*) mg of catechin equiv./100 g of FW. *B. penduliflora* leaves had the highest total flavonoid (1682.4±27.7 mg of CAE/100 g of FW). *B. penduliflora* leaves had the highest free flavonoid (1229.81±75.78 mg of CAE/100 g of FW), up to 73.10% of total flavonoid, and had the highest bound phenolics (452.63±57.1 mg of CAE/100 g of FW), accounted for 26.9% of total flavonoids.

### Phenolic acid compounds in ramie leaves

The concentrations of represented phenolic acid, chlorogenic acid, caffeic acid, 4-coumaric acid, ferulic acid, and benzoic acid, were analyzed by HPLC and the results are shown in Table 1. Chlorogenic acid was in not detected in all samples of bound form, and was detected in two samples of free form, *B. nivea* (38.24±2.15 mg/100 g), *B. tricuspis* (30.19±0.05 mg/100 g). Caffeic acid was not detected in all samples of free form. *B. tricuspis* had the highest bound caffeic acid (25.79±0.01 mg/ 100 g), followed by *B.* nivea (18.38±0.02 mg/100 g), *B. macrophylla* (12.04±0.01 mg/100 g), *B. longispica* (4.81±0.01 mg/100 g) and *B. clidemioides*(0). *B. nivea* had the highest bound 4-coumaric acid (9.77±0.12 mg/100 g), followed by *B. tricuspis* (7.33±0.67 mg/100 g), *B. macrophylla* (4.60±0.001 mg/100 g), *B. longispica* (3.74±0.05 mg/100 g), and *B. clidemioides* (2.46±0.1 mg/100 g). Ferulic acid was not detected in all samples of free form. *B. longispica* had the highest bound ferulic acid (21.33±0.21 mg/100 g), followed by *B. penduliflora* (18.76±1.34 mg/100 g), *B. nivea* (13.63±0.17 mg/100 g), *B. macrophylla* (11.22±0.13 mg/100 g), *B. tricuspis* (6.43±0.38 mg/100 g), and *B. clidemioides* (5.28±0.17 mg/100 g). *B. nivea* had the highest bound benzoic acid (28.9±1.29 mg/100 g), followed by *B. tricuspis* (25.88±6.28 mg/100 g), *B. penduliflora* (22.31±1.73 mg/100 g), *B. longispica* (20.76±0.72 mg/100 g), *B. macrophylla* (16.67±0.02 mg/100 g), and *B. clidemioides* (10.98±1.85 mg/100 g). *B. tricuspis* had the highest free benzoic acid (863.22±1.44 mg/100 g), followed by *B. nivea* (151.67±1.20 mg/100 g), *B. clidemioides* (60.95±0.19 mg/100 g), and *B. longispica*, *B. macrophylla* were not detected. *B. tricuspis* had the highest total benzoic acid (889.1±9.66 mg/100 g), followed by *B. nivea* (180.57±1.11 mg/100 g), *B. clidemioides* (71.93±1.62 mg/100 g), *B. penduliflora* (22.31±1.73 mg/100 g), *B. longispica* (20.76±0.72 mg/100 g), and *B. macrophylla* (16.67±0.02 mg/100 g).

**Table 1:** Phenolic compounds in six species of ramie leaves.

		*B. Nivea*	*B. Longispica*	*B. Clidemioides*	*B. Macrophylla*	*B. Tricuspis*	*B. Penduliflora*
Chlorogenic acid (mg/100 g FW)	bound	ND	ND	ND	ND	ND	ND
	free	38.24±2.15	ND	ND	ND	30.19±0.05	ND
	total	38.24±2.15	ND	ND	ND	30.19±0.05	ND
Caffeic acid (mg/100 g FW)	bound	18.38±0.02	4.81±0.01	ND	12.04±0.01	25.79±0.01	24.78±0.04
	free	ND	ND	ND	ND	ND	ND
	total	18.38±0.02	4.81±0.01	ND	12.04±0.01	25.79±0.01	ND
4-coumaric acid (mg/100 g FW)	bound	9.77±0.12	3.74±0.05	2.46±0.10	4.6±0.001	7.33±0.67	5.86±0.65
	free	ND	10.43±0.92	ND	ND	ND	ND
	total	9.77±0.12	14.17±1.00	2.46±0.10	4.60±0.001	7.33±0.67	5.86±0.65
Ferulic acid (mg/100 g FW)	bound	13.63±0.17	21.33±0.21	5.28±0.17	11.22±0.13	6.43±0.38	18.76±1.34
	free	ND	ND	ND	ND	ND	ND
	total	13.63±0.17	21.33±0.21	5.28±0.17	11.22±0.13	6.43±0.38	18.76±1.34
Benzoic acid (mg/100 g FW)	bound	28.90±1.29	20.76±0.72	10.98±1.85	16.67±0.02	25.88±6.28	22.31±1.73
	free	151.67±1.20	ND	60.95±0.19	ND	863.22±1.44	ND
	total	180.57±1.11	20.76±0.72	71.93±1.62	16.67±0.02	889.10±9.66	22.31±1.73

ND: not detected. FW: fresh weight.

### *In vitro* antioxidant activity of ramie leaves

**Figure 3:**
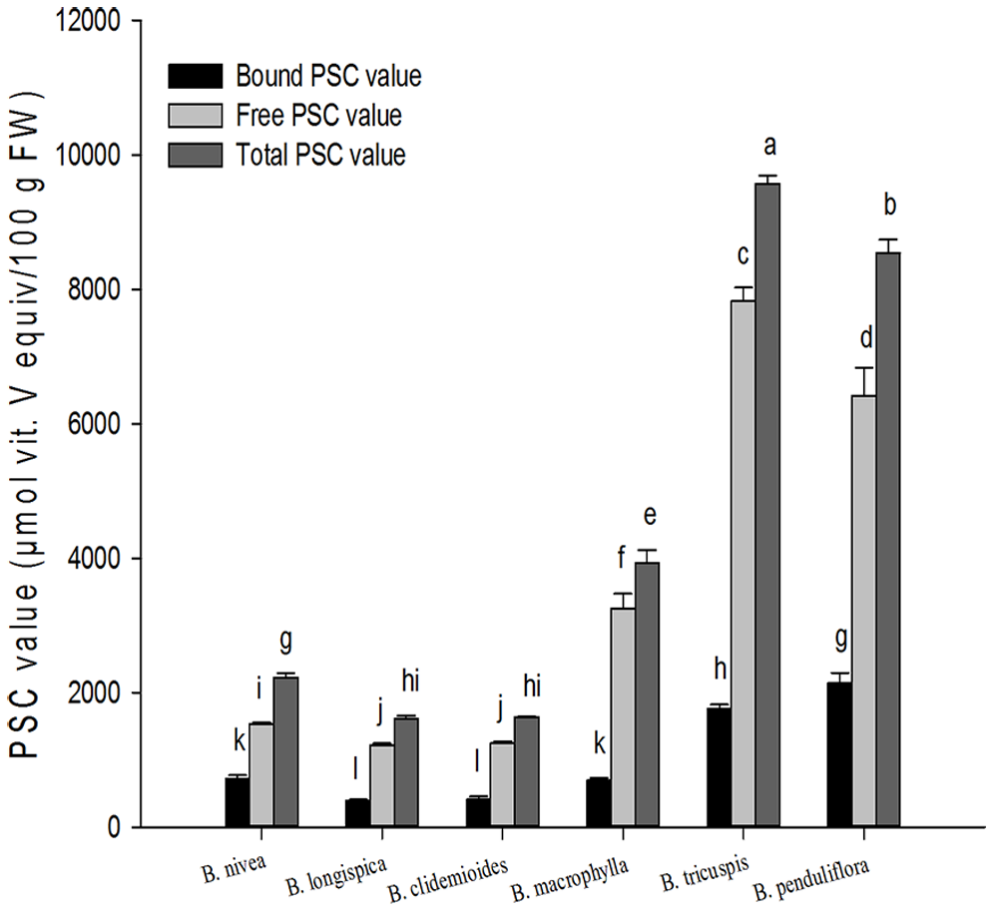
PSC values in six species of ramie leaves (mean ± SD, n = 3). Bars with no letters in common are significantly different (*p*<0.05).

Owing to the complex reactivity of phytochemicals, the antioxidant activities were evaluated using two methods, ORAC and PSC. The PSC values of ramie leaves are presented in Figure 3, expressed as micromoles of vitamin C equivalents per 100 g of fresh weight. The free PSC values ranged from 1216.7 (*B. longispica*) to 7827.9 (*B. tricuspis*) µmol of vitamin C equiv./100 g FW. The bound PSC values ranged from 394.48 (*B. longispica*) to 2139.2 (*B. penduliflora*) µmol of vitamin C equiv./100 g FW. The total PSC values ranged from 1611.2 (*B. longispica steud*) to 9574.8 (*B. tricuspis*) µmol of vitamin C equiv./100 g FW. *B. tricuspis* had the highest total hydrophilic peroxyl radical scavenging capacity among the six species of ramie, which was 6 times higher than the lowest (*B. longispica*), followed by *B. penduliflora*, *B. macrophylla*, *B. nivea*, *B. clidemioides* and *B. longispica* had the lowest antioxidant activity among the varieties tested.

**Figure 4:**
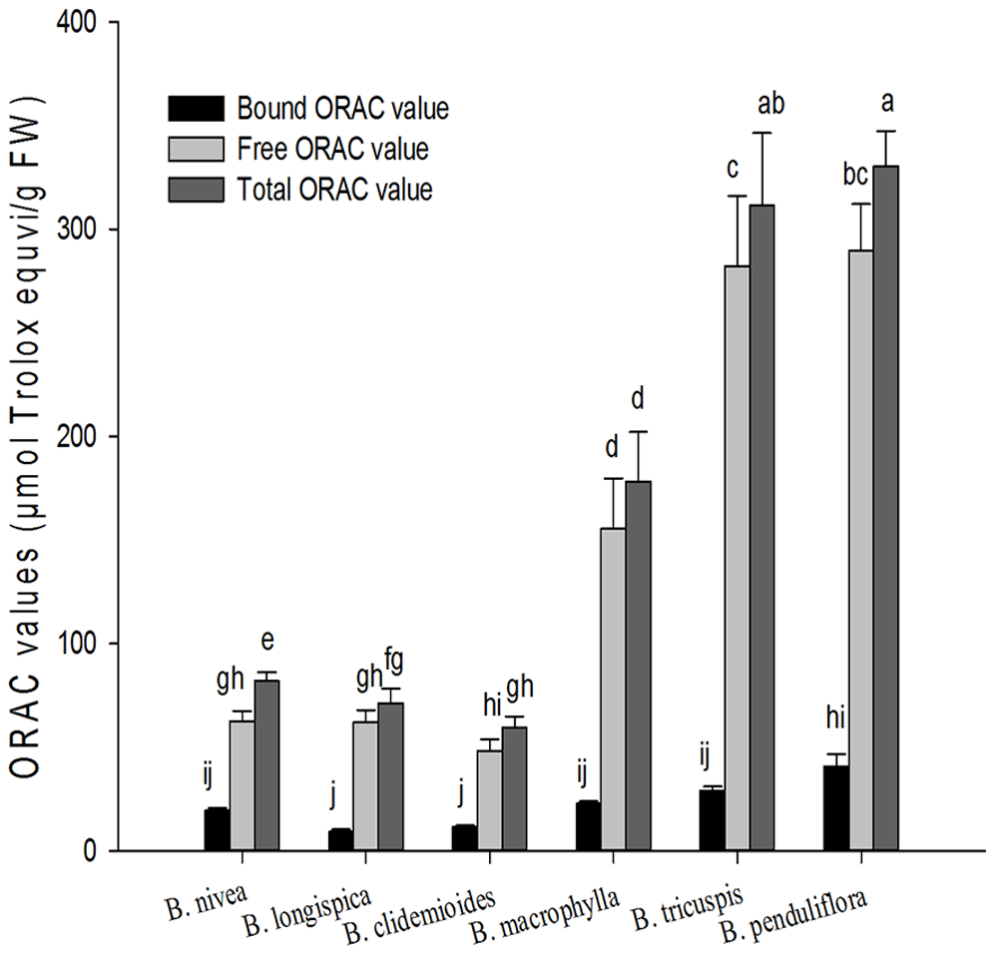
ORAC values in six species of ramie leaves (mean ± SD, n = 3). Bars with no letters in common are significantly different (*p*<0.05).

The ORAC values of ramie leaves are presented in Figure 4, expressed as micromoles of Trolox equivalents per g of fresh weight. The free ORAC values ranged from 62.04 (*B. longispica*) to 289.57 (*B. penduliflora*) µmol of Trolox equiv./g FW. The bound ORAC values ranged from 9.20 (*B. longispica*) to 40.87 (*B. penduliflora*) µmol of Trolox equiv./g FW. The total ORAC values ranged from 59.58 (*B. clidemioides*) to 330.44 (*B. penduliflora*) µmol of Trolox equiv./g FW. *B. penduliflora* had the highest total peroxyl radical scavenging efficacy among the six species of ramie, which was 6 times higher than the lowest (*B. clidemioides*), followed by *B. tricuspis*, *B. macrophylla*, *B. nivea*, *B. longispica*.

### *In vivo* cellular antioxidant activities in ramie leaves

The cellular antioxidant activities of the ramie leaves extracts were measured using the CAA assay. The cellular antioxidant activities were measured using two protocols (PBS wash and no PBS wash). The EC_50_ values ranged from 6.70 (*B. tricuspis*) to 74.54 (*B. nivea*) mg/mL in the PBS wash protocol and from 2.24 (*B. tricuspis*) to 36.08 (*B. clidemioides*)mg/mL in no PBS wash protocol. In both methods, the *B. tricuspis* had the lowest EC_50_ values (6.70±0.36 in the PBS wash protocol and 2.24±0.18 mg/mL in the no PBS wash protocol). The *B. nivea* had the highest EC_50_ values (74.54±16.84 mg/mL) in the PBS wash protocol, followed by *B. longispica* (72.21±9.01 mg/mL), *B. clidemioides* (66.54±9.65 mg/mL), *B. macrophylla* (14.77±1.32 mg/mL), *B. penduliflora* (11.23±0.59 mg/mL), *B. tricuspis* (6.70±0.36 mg/mL). The *B. clidemioides* had the highest EC_50_ values 36.08±5.50 mg/mL) in the no PBS wash protocol, followed by *B. longispica* (18.20±2.63 mg/mL), *B. nivea* (11.17±1.53 mg/mL), *B. macrophylla* (6.14±0.99 mg/mL), *B. penduliflora* (3.76±0.29 mg/mL), *B. tricuspis* (2.24±0.18 mg/mL).

**Figure 5:**
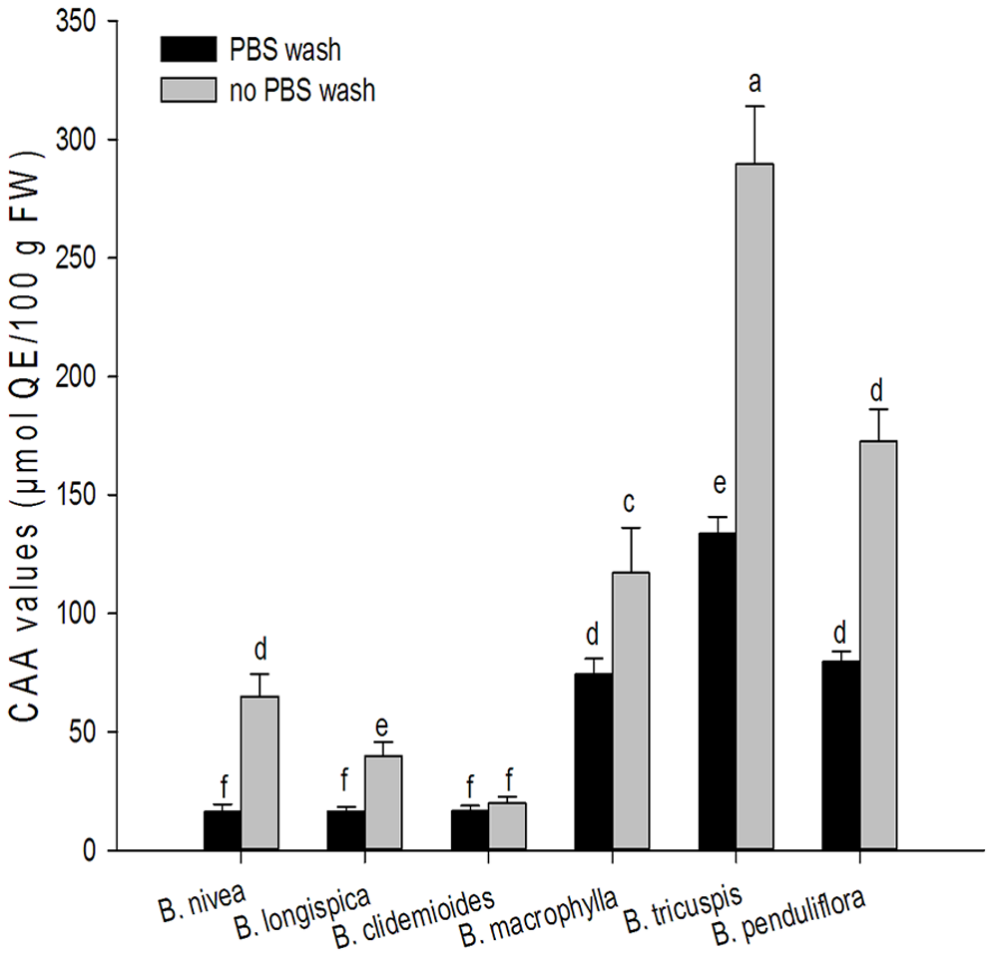
CAA values in six species of ramie leaves (mean ± SD, n = 3). Bars with no letters in common are significantly different (*p*<0.05).

We reported the CAA values of ramie leaves firstly. The CAA values for ramie leaves extracts were showed in Figure 5. CAA values ranged from 16.27 (*B. nivea*) to 133.63 (*B. tricuspis)* µmol QE/100 g FW of ramie in the PBS wash protocol. The *B. tricuspis* had the highest CAA value (133.63±7.10 µmol QE/100 g FW of ramie), followed by *B. penduliflora* (79.70±4.27 µmol QE/100 g FW of ramie), *B. macrophylla* (74.30±6.55 µmol QE/100 g FW of ramie), *B. clidemioides* (16.63±2.27 µmol QE/100 g FW of ramie), *B. longispica* (16.45±1.91 µmol QE/100 g FW of ramie), *B. nivea* (16.27±3.28 µmol QE/100 g FW of ramie). In the no PBS wash protocol, CAA values ranged from 19.88 to 289.60 µmol QE/100 g FW of ramie, *B. tricuspis* had the highest CAA value (289.60±24.35 µmol QE/100 g FW of ramie), followed by *B. penduliflora* (172.59±13.64 µmol QE/100 g FW of ramie), *B. macrophylla* (117.10±19.06 µmol QE/100 g FW of ramie), *B. nivea* (64.92±9.40 µmol QE/100 g FW of ramie), *B. longispica* (39.89±5.91 µmol QE/100 g FW of ramie), *B. clidemioides* (19.88±2.79 µmol QE/100 g FW of ramie).

The CAA and EC_50_ values of the different ramie extracts were negatively correlated, the lower the EC_50_ value, the higher the CAA value. Regardless of the protocol, *B. tricuspis* had the lowest EC_50_ value.

### Antiproliferation activity and cytotoxicity in ramie leaves

The inhibition of HepG2 cell proliferation in vitro by the ramie leaves extracts and the cytotoxic effects are presented in and Table 2. The results were expressed as the median effective dose (IC_50_), with a lower IC_50_ value indicating a higher antiproliferation activity. The six ramie leaves extracts showed potent inhibit cell proliferation on HepG2 cell growth in a dose-dependent manner, and there were differences among the six ramie extracts. The extracts of *B. tricuspis* had the highest inhibitory effects on cell proliferation, *B. longispica* showed the lowest antiproliferative activity. *B. penduliflora* had cytotoxic effects (CC_50_≥1.15 mg/mL), which showed that the inhibitory effect was due to cytotoxic effect. But others had no cytotoxic effects in the proliferation of concentration, which indicated that the inhibitory effect was attributed to antitumor effects of the extracts.

**Table 2:** Antiproliferative Activities (IC_50_) and Cytotoxicities (CC_50_) of Ramie Leaves Extracts toward Human HepG2 Cancer Cells.

	IC_50_(mg/mL)	CC_50_ (mg/mL)
*B. longispica*	48.06±5.81a	>20
*B. macrophylla*	6.11±0.03c	>11.9
*B. clidemioides*	13.85±1.82b	>20
*B. tricuspis*	4.11±0.19c	>11.3
*B. penduliflora*	1.46±0.11c	>1.15
*B. nivea*	ND	>20

Values with no letters in common in each column are significantly different (*p*<0.05). ND: not detected.

## Discussion

Ramie leaves as food and traditional medicine has a long history. Different varieties of Ramie leaves are not only good sources for dietary and medical use, but also excellent sources of phytochemicals, such as phenolics and flavonoids.

Phenolics compounds may provide health benefits related to decrease the incidence of chronic disease in our diet. This is first report of phenolics in fresh leaves of ramie indicated that phenolics in soluble free forms were significantly higher than bound forms in all selected ramie. Phenolics and flavonoids are present in free and bound (cell wall-associated) forms in plants. We developed a detection method to measure the complete phenolic profiles, this method identified and quantifies the free and bound forms of phenolics [4]. According to the present study, phenolics is one of the main phytochemical in ramie leaves.

Similarly to phenolics, contents of flavonoid in soluble free form were significantly higher than bound flavonoid contents in all selected ramie. Flavonoids, widespread in teas, fruits, vegetables and medicinal plants, are a group of biologically active plant compounds [1], [5]. They were also reported to exhibit antioxidant activity and could inhibit cellular growth [21], [22]. Ramie, widely cultivated in China, is a flavonoid rutin rich plant source [23]. There is no published report of flavonoids in ramie leaves. This study was the first report to quantify total flavonoid contents in fresh leaves of ramie using a new method reported recently [13]. The SBC assay, developed by our group, can quantify all types of flavonoids including all subgroups of flavones, flavonones, flavononols, flavonols, flavanols, isoflavonoids and anthocyanins [14]. The occurrence of phenolic acid in food affects stability, color, flavoe, nutritional value, and other qualities. Phenolic acids (*p*-coumaric acid, caffeic acid, ferulic acid and ellagic acid) have been proposed to have beneficial effects on health as antioxidants [24]. The potential of using natural phenolic acid as natural antioxidants has drawn more attention. Ferulic acid, arises from the metabolism of phenylalanine and tyrosine [25], is a ubiquitous plant component also process some biological activities [6]. Ferulic acid is mainly based on the free form and bound in seeds and leaves. Ferulic acid serves an vital antioxidant function in preserving physiological integrity of cells, its addition of foods inhibits lipid peroxidation and subsequent oxidative spoilage [25]. Humans ingest a variety of phenols. Caffeic acid, chlorogenic acid, ferulic acid suppressed the promotion of tumors [26], [27]. Caffeic acid has free radical scavenging property and the effect of inhibiting LDL oxidative [28]. To test if the high antioxidant activity of ramie was attributable to the phenolic acid, we analyzed the main phenolic acid. The results reported in this article demonstrated that phenolic acids were showed in bound form, and were in agreement with the reported by S. C. Fry [29].

Various phytochemical components, such as phenolic acids, flavonoids, are contributed to antioxidant capacity in fruits and vegetables [1], [5], and have attracted interest because of their potential nutritional and safety. Due to the complex reactivity of phytochemicals, the accurate evaluate of antioxidant activities of phytochemicals to be taken into account. It is suggested that the use of more than one condition of oxidation is required to evaluate antioxidants. In this study, the antioxidant activities were evaluated using two methods, named ORAC and PSC.

There are many kinds of methods detect antioxidant activity. One of these methods is named PSC assay which incorporating dichloroflurescin diacetate (DCFH-DA) as a fluorescent probe to monitor reaction, and is suitable for analyzing both hydroplic and lipophilic antioxidants or food extracts [2]. PSC method is reliable, sensitive, rapid, reproducibility, and precise and can produce acceptable results compare with those obtained with similar published assays [2]. Oxygen radical absorbance capacity (ORAC) assay has been widely accepted as a tool to test the antioxidant activity in the pharmaceutical and food industries. In the typical ORAC assay, the fluorescent loss of probes as phycoerythrin, and then fluorescein is followed kinetics in the absence and presence of antioxidant [30]. The antioxidant activity of *Nivea* extract has been evaluated using DPPH radical scavenging activity [31]. However, it is difficult to compare their results to the antioxidant activity reported here using the PSC/OARC assay. In accordance with previous report that the antioxidant activity of extracts of blueberries were due to phenolic compounds in these berries [32], we reported significant linear relationship between phenolic/flavonoid contents and PSC/ORAC values.

All percent contribution of bound phytochemicals to total were no more than 45%, most percent contribution of bound phytochemicals were about 20%, it was hard to analyze the antioxidant activities in bound phytochemicals, so in cell we used free phytochemicals for analysis.

Cellular antioxidant activity (CAA) assay, quantifying the antioxidant activity of phytochemicals, food extracts, and dietary supplements, perform at physiological pH and temperature, and take into account the bioavailability, uptake, and metabolism of the antioxidant compounds [18]. Cell culture models are cost-effective, relatively fast, and better represent the complexity of biological systems.

The increase in fluorescence from the formation of DCF was inhibited by both quercetin and ramie extracts in a dose-dependent manner. DCFH oxidation was inhibited regardless of whether the cells had been washed with PBS or not between the antioxidant and the ABAP treatments. Compared to the PBS wash protocol, the no PBS wash protocol had significantly lower EC_50_ values and higher antioxidant capacity. This result is attributed to the PBS, which can affect the extracellular antioxidant capacity, thereby reducing the intracellular antioxidant capacity.

The correlations between phenolic content, flavonoid content and antioxidant activity were examined. Significant linear relationship was found between phenolic/flavonoid contents and PSC values of different ramie types (R^2^ = 0.943, *p*<0.05; R^2^ = 0.771, *p*<0.05;). A significant linear relationship was found between phenolic/flavonoid content and ORAC values of different ramie types (R^2^ = 0.943, *p*<0.05; R^2^ = 0.771, *p*<0.05). A highly significant linear relationship was found between phenolic/flavonoid contents and CAA values (PBS wash) of different ramie types(R^2^ = 0.943, *p*<0.01; R^2^ = 0.943, *p*<0.01). Significant linear relationship was also found between phenolic/flavonoid contents and CAA values (no PBS wash) of different ramie types (R^2^ = 0.714, *p*<0.05; R^2^ = 0.714, *p*<0.05;). The positive correlation shows that antioxidant activities were closely related to phenolics and flavonoids in six species of ramie.

To estimate the effect of ramie leaves on cancer cell growth, we assessed its effect on the proliferation of liver cancer cells. According to our data, ramie leaves extract showed inhibit proliferation activity, the mechanism may be ramie leaves extract enhance anti cancer cytokines activity. In contrast to data, *B. Penduliflora* and B. Tricuspis makino is similar to phytochemical prolifes, however, *B. Penduliflora* showed lowest IC_50_ and CC_50_, the data suggested that the mechanisms of *B. Penduliflora* inhibit proliferation is associated with apoptosis.

In summary, we found that the primary portion of phytochemicals in the fresh leaves of ramie was showed in the free form, intimating that most phytochemical of fresh leaves of ramie is soluble. There are significant differences in phytochemical content and antioxidant activity among the different varieties of ramie leaves. However, some similarities can be seen within families and genera. Ramie leaves are the potential sources for food and pharmaceutical applications as anticancer agents. However, further research is needed to evaluate the effects of ramie leaf in the prevention of liver cancer.
